# Using precision phenotyping to inform de novo domestication

**DOI:** 10.1093/plphys/kiab160

**Published:** 2021-04-12

**Authors:** Alisdair R Fernie, Saleh Alseekh, Jie Liu, Jianbing Yan

**Affiliations:** 1 Max Planck Institute for Molecular Plant Physiology, Am Mühlenberg 1, 14476 Potsdam-Golm, Germany; 2 Centre of Plant Systems Biology and Biotechnology, 4000 Plovdiv, Bulgaria; 3 National Key Laboratory of Crop Genetic Improvement, Huazhong Agricultural University, 430070 Wuhan, Hubei, China

## Abstract

An update on the use of precision phenotyping to assess the potential of lesser cultivated species as candidates for de novo domestication or similar development for future agriculture.

## Introduction

It is an oft-cited fact that toward the middle of this century, the rate of increase in global population is likely to outstrip that in global agricultural production. Moreover, productivity varies greatly across the globe, yet the majority of the burden on agriculture is placed on the cultivation of a small number of species largely in locations different from their origin of domestication and often subject to far different environmental conditions (Fernie and Yan, 2019). Recent technical developments—mainly the enhanced accessibility and affordability of next-generation sequencing technologies—have allowed the identification of over 100 domestication genes (Fernie and Yan, 2019). Many of these, such as those associated with the loss of shattering, seed size, and dormancy, are conserved across our crop species (Gross and Olsen, 2010; Lenser and Theissen, 2013). However, others seem to be specific to certain crops or crop types such as the modification of fruit shape (Xiao et al., 2008) or the evolution of tubers (Cheng et al., 2016; Hardigan et al., 2017). Having identified the genes, they can be used for de novo domestication, that is the genetic improvement of little cultivated or as yet undomesticated species. Key to this is the identification of species that display specific desired properties, for example higher production and fertilizer use efficiency, more balanced nutritional properties, better flavor or, alternatively, that are better suited for growth under certain agronomic conditions. To date, a handful of examples of de novo domestication have been published, which act as a proof-of-concept of the approach (Lemmon et al., 2018; Li et al., 2018; Zsögön et al., 2018; Hoyos et al., 2020; Yu et al., 2021). Moreover, several projects such as The African Orphan Crops Consortium (Jamnadass et al., 2020) have been initiated, which aim to use this approach at a very large scale.

**Advances box**

- Recent years have seen an explosion in the number of domestication alleles that have been demonstrated to underlie important agronomic traits.
- Gene editing techniques allow the manipulation of (multiple) such alleles into as yet undomesticated species as a method to produce crops better suited to a given environmental niche.
- Precision phenotyping approaches are beginning to be used in the design and execution of such projects and will undoubtedly become a key aspect of such endeavors.

This Update focuses on recent progress made in both de novo domestication per se and in the use of precision phenotyping to assess suitable candidates for the process. Largely, but not exclusively, using teosinte-maize (*Zea mays*) as a case-study we (i) detail how computational methods can be used for identifying domestication genes and evaluating changes associated with these genes at both the phenotypic and molecular levels (Broman et al., 2019), (ii) demonstrate the power of precision phenotyping in evaluating the potential phenotypic scope of such interventions (Yang et al., 2020), (iii) describe the combined power of bioinformatics approaches and CRISPR/Cas libraries for streamlining agricultural engineering strategies (Childs et al., 2012; Liu et al., 2020), (iv) explain the benefit of harnessing recently acquired information concerning natural structural variations in crop and close-relative genomes (Alonge et al., 2020; Domínguez et al., 2020), (v) outline the potential role single cell sequencing will play in applying these methods to polyploid species (Luo et al., 2020), and finally (vi) discuss the prospects of recent approaches integrating molecular markers into metabolic models to improve genome selection (Tong et al., 2020). We discuss in detail the concepts underlying these approaches rather than the technical detail of either next-generation sequencing, genome editing, or plant phenotyping studies, which have been the subjects of recent reviews in their own right (Schneeberger and Weigel, 2011; Pacher and Puchta, 2017; Yang et al., 2020).

## De novo domestication—the state of the art

The combination of global climate change, our expanding population and the increasing growth of plants for biofuel and other non-food purposes is leading to an ever increasing demand for agronomic efficiency. Indeed, productivity varies greatly across the globe and while the production efficiency of cereals has kept pace with the human population, this is not true for all crops and some are even poorly suited to their area of cultivation. For example, the yields of cassava (*Manihot esculenta*) are three times higher in South East Asia than in Africa (Sonnewald et al., 2020). This instability aside, we only cultivate around 150 species to a large extent and 70% of the calories consumed by humans comes from only 15 of these species (Fernie and Yan, 2019). That said, more than 7,000 of the 400,000 extant plant species are regarded as semi-cultivated (Smýkal et al., 2018) and could represent important germplasm for the design of future crops. Here, we describe already published examples of plant de novo domestication before briefly outlining what makes a good candidate species for this approach. Following this introduction, we detail the integral role we envisage computational and precision phenotyping will play in future strategies of de novo domestication and re-domestication.

Four recent studies in the Solanaceae demonstrate the potential of gene editing for de novo domestication. Two of the studies included the orphan crop groundcherry (*Physalis pruinosa*; Lemmon et al., 2018; Kwon et al., 2020), while three included de novo domestication of the wild tomato *Solanum pimpinellifolium* or alteration of the MicroTom cutivar (Li et al., 2018; Zsögön et al., 2018; Kwon et al., 2020). The studies on groundcherry included development of a transformation procedure alongside the development of genomic resources for the species. Following the acquisition of this competence, the authors first knocked out *SELF-PRUNING* (*SP*) and *SELF-PRUNING-5G* (*SP-5G*) with the latter resulting in enhanced auxiliary flowering and increased fruit density, as well as targeting the *CLAVATA* (*CV*) pathway, which increased floral meristem size and led to additional floral organs (Lemmon et al., 2018). Analogous studies in *S. pimpinellifolium* targeted these three, and a further eight, genes (Li et al., 2018; Zsögön et al., 2018). They also proved successful in altering target traits, for example, doubling the yield of *S. pimpinellifolium* and enhancing lycopene levels five-fold. In the fourth study, both the MicroTom cultivar of *S. lycopersicum* and groundcherry were modified either by stacking a gene for tomato stem length with *SP5G* or *SP* or targeting the same stem length regulator alone. Both approaches led to a compact stature and early yielding plants suitable for urban agriculture (Kwon et al., 2020). Although we note that the work on MicroTom is clearly not an example of de novo domestication per se that on groundcherry is. Actually, the term de novo domestication is not universally accepted, given that domestication over millennia is acting on the whole genome in concert (Stetter et al., 2018; Stetter, 2020). While understanding these concerns, it is a term that has been rapidly adopted and a clear consensus concerning its meaning has been reached. In essence, de novo domestication refers to a deliberate modification of the sequence of a domestication gene in a lesser grown species with the aim of producing an agriculturally useful plant.

Although many of the early examples of de novo domestication have been carried out in the Solanaceae, the approach is by no means limited to this family, with examples in pennycress (*Thlaspi arvense* L.; McGinn et al., 2019), sunflower (*Helianthus annuus*; Ekar et al., 2019; Van Tassel et al., 2020), and the legume *Vigna stipulacea* (Takahashi et al., 2019). Pennycress has been de novo domesticated as a seed cover crop for the winter fallow period. For this purpose, CRISPR was used to produce insertion/deletion (indel) mutations in the *FATTY ACID ELONGATION1* (*FAE1*) gene, thereby abolishing erucic acid production and creating an edible seed oil comparable to that of canola (McGinn et al., 2019). By contrast, conventional breeding has been used to domesticate the oilseed *Silphium*, resulting in increased aboveground biomass at the seedling and adult stages and a greater increase in seed yield, combining to a modest improvement in harvest index (Van Tassel et al., 2020). A similar approach has been utilized to follow sunflower domestication in order to develop a perenial crop that can produce both high value vegetable oil and continuous ground cover (Ekar et al., 2019). Using an alternate approach, Takahashi et al. (2019) carried out ethyl methanesulfonate (EMS) mutagenesis and screening of a *V. stipulacea* population, isolating mutants with reduced seed dormancy and shattering, respectively. *Vigna* represents an interesting legume species for de novo domestication given that it exhibits rapid growth, a short vegetative stage, and broad resistance to pests and diseases. Although it will be quite time-consuming, the authors postulate that by pyramiding these mutant phenotypes, they will be able to generate a primitive crop which can be cultivated without pesticide. In addition to these specific examples, considerable research effort is also ongoing in close relatives of sorghum (*Sorghum bicolor*) and sugarcane (*Saccharum officinarum*; Paterson et al., 1995a, 1995b; Zhang et al., 2018) and the cases for utilizing many other crop wild relatives not least for their enhanced stress tolerance have been convincingly made (Zhang et al., 2018; Fernie and Yan, 2019).

A recent tour-de-force of the de novo domestication of the allotetraploid wild rice *Oryza alta* was published in Cell (Yu et al., 2021). In this article, a breeding route was presented in order to harness the advantages polyploidization in terms of genome buffering, vigorousness, and environmental robustness to rice. To do so, the authors developed an efficient transformation system, thus facilitating gene editing and a high-quality genome assembly of *O. alta*. In this example, *O. alta* was chosen after evaluating three wild species (8 *O. alta*, 2 *Oryza grandiglumis*, and 18 *Oryza latifolia* lines) and selecting *O. alta* due to its callus induction and regeneration properties. Following this, as a case study, six agronomically important traits, namely shattering, awn length, hull color, pericarp color, panicle shape, and grain width, were rapidly improved in *O. alta*, thereby demonstrating the feasibility of its de novo domestication. This study is arguably the first true de novo domestication and illustrates the importance of developing transformation systems and genome sequences as enabling steps for this process.

## Accelerating the identification of domestication genes

As stated above, rapid de novo domestication requires both genomic information and effective transformation procedures. Similarly, the alternative genetics-based approaches of crossing near relatives in attempt to introgress a trait of interest or the adoption of mutagenesis-based strategies have been greatly facilitated by the generation of computational tools that render them considerably easier (Huang and George, 2011; Youens-Clark et al., 2011; Meng et al., 2015; Broman et al., 2019; Wei et al., 2021). However, given that all of these have become relatively standard laboratory techniques, we discuss only the utility of genome data in the identification of domestication genes here.

**Figure 1:**
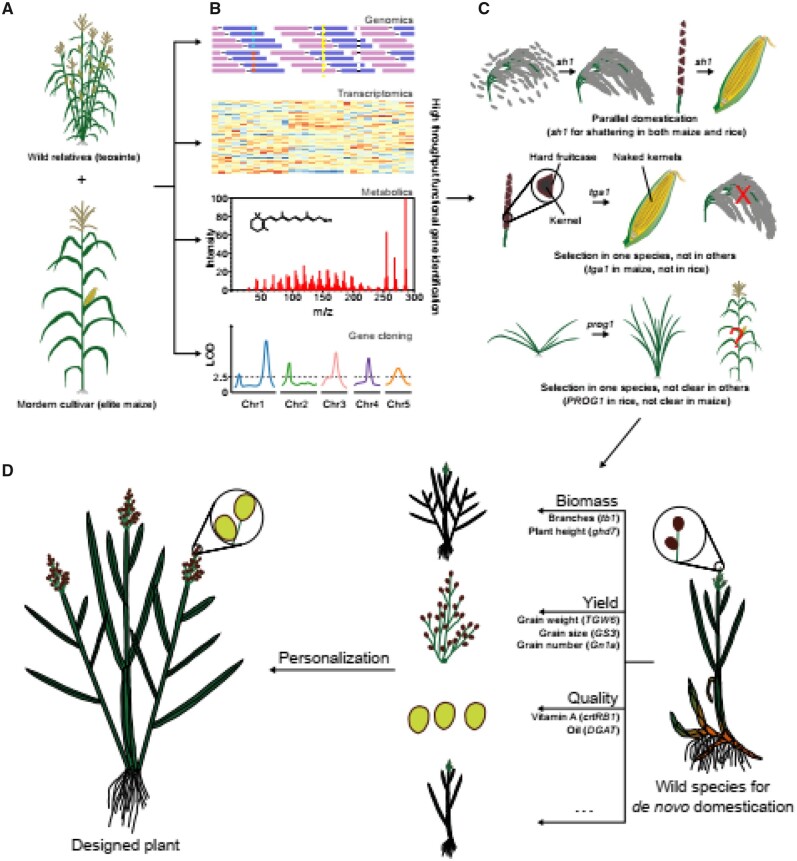
Overview of knowledge-driven de novo domestication. A, The wild relatives and modern cultivars are vital germplasms (e.g. teosinte and maize) for tracing selection signals during plant domestication and also functional genomics research. B, Multiple-omics, including genomics, transcriptomics, metabolomics, and phenomics, are foundations for high-throughput gene cloning and gene function analysis. C and D, Wild species chosen for de novo domestication could be quickly shaped to meet different kinds of demands through selections on detected signals during previous domestications, especially parallel selected genes. Ideal plants can thus be designed through the pyramiding and modification of genes of diverse function.

The use of multiomics in de novo domestication is summarized in Figure 1, which we first describe here before dissecting the various layers involved in detail in the following paragraphs. Wild relatives and modern cultivars as exemplified by teosinte and maize (a comparison we routinely use, since, as mentioned above, it arguably represents the best characterized material covering plant domestication and improvement; Figure 1, A) are highly important for tracking signals of selection and thereby defining the genomic regions underlying the phenotypic changes during the process. Comparisons of the progeny of a biparental cross between the wild and cultivated species are commonly made, subjected to metabolomics and transcriptomics, and the underlying quantitative trait loci including domestication genes are cloned (Figure 1, B). Literature information concerning the roles of these genes in other species can then be mined, since where they were able to provide clear answers; previous studies have revealed that such genes are either subject to parallel domestication or species-specific domestication events (Figure 1, C). Following such assessments traits of interest, such as biomass, yield, and quality, can be modified via gene editing of the underlying genes allowing de novo domestication and personalization of the de novo domesticate (Figure 1, D).

In practice, the tracking of signals of selection is carried out by computational analyses known as selective sweeps, which are essentially a measure of the genetic diversity between cultivated and ancestral genotypes. Following this approach, it is assumed that those regions that have been selected for during domestication harboring less diversity than those that regions that are subject to less stringent selection (Zhu et al., 2018). When a genetic variant increases its frequency due to positive selection, the adjacent alleles also increase in their frequency in a process termed hitchhiking (Smith and Haigh, 2007). When the genetic variant under selection reaches high frequency (or even fixation), the hitchhiking effect reduces (or even eliminates), the genetic variation around the selected locus in an effect known as a selective sweep (Pavlidis and Alachiotis, 2017). Local *F*_ST_ outlier tests can be used to detect selective sweeps between populations of wild and domesticated taxa (Gepts, 2014). There are many different methods based on the *F*_ST_ statistic, which largely vary in the underlying model used to calculate the null distribution of *F*_ST_ values (see e.g. Bonhomme et al., 2010; Lotterhos and Whitlock, 2015). Selecting a suitable method is complicated, with considerations including the sampling scheme taken, the total size of the dataset, intensity of selective pressure, and the genetic structure of the population all needing to be taken into account (Barrera-Redondo et al., 2020). Illustrative examples of the use of these tests in plants include the detection of domestication genes involved in apple (*Malus domestica*) fruit development, size, acidity and sugar content (Khan et al., 2014), size, color, and disease resistance in tomato (Zhu et al., 2018), and those for oil biosynthesis in sunflower (Baute et al., 2015).

Given the torrent of genome sequence information, it seems likely that optimization of computational approaches will greatly accelerate identification of domestication genes. Indeed, if pedigree is established from genome information and this allows clear discrimination of crop wild relatives, landraces, and cultivars, similar experiments can be carried out to define domestication and improvement genes for many species. It is worth noting that a mere 15 years ago the number of confirmed domestication genes numbered just over two dozen (Doebley et al., 2006); however, there are now well over a hundred with notable recent genes being identified for dormancy in rice, soybean (*Glycine max*), and tomato (Wang et al., 2018), the evolution of tubers in for example potato (Hardigan et al., 2017) and grain filling in maize (Sosso et al., 2015). Many of the genes involved are transcription factors (Fernie and Yan, 2019) as would perhaps be anticipated given the dramatic upregulation of gene expression on domestication evidenced in teosinte to maize comparisons (Swanson-Wagner et al., 2012). Beyond searching genomes for signatures of selection, detailed characterization of the transcriptome, metabolome, and phenome will represent important information when choosing which species is most appropriate for de novo domestication. We discuss the first two of these here, but the third is so important that it warrants its own section below. Before we do so, however, it should be noted that the aim of all of these is a broad characterization of lesser studied species rather than merely an assessment of the variance of a trait of interest. Put another way, the domestication genes already described are the likely tools for de novo domestication but deciding the likely species to domesticate first requires a better understanding of the physiology of a range of species. One could, for example, look for species that thrive in a particular environment, have a nutritionally excellent chemical composition, high nitrogen or water use efficiencies, or are naturally pest-resilient. That said, we certainly appreciate that de novo domestication will be easier for similar species, such as the excellent examples in rice and the Solanaceae. A major question for the future is how easy it will be to transfer allelic diversity between members of different genera, tribes, or even families where there are as yet no domesticated species. It is too early to answer this question. Many domestication traits are shared by a very wide number of species, while others are more taxonomically restricted (Meyer et al., 2012). This clearly renders de novo domestication of completely wild species that are taxonomically distant from our major crops exceptionally hard. By contrast, those traits which are widely shared represent obvious targets; however, that said, even if de novo domestication is restricted to crop wild relatives, it is likely to allow great agronomic advances.

Transcript profiles are tissue-specific and subject to temporal control, thus transcriptomics can reveal important loci involved in domestication traits such as suppression of secondary metabolites, changes in form, size, taste, absence of defense mechanisms, seed dormancy, and many other traits (Barrera-Redondo et al., 2020). For example, the assessment of altered gene expression on domestication was achieved in maize, tomato, lettuce (*Lactuca sativa*), and common bean (*Phaseolus vulgaris*) by comparing the total gene expression of a range of wild species, progenitors, and cultivars, respectively (Swanson-Wagner et al., 2012; Koenig et al., 2013; Bellucci et al., 2014). A comparison of 38 diverse maize and 24 teosinte provided evidence for more than 600 genes having significantly different expression levels and almost twice as many altered co-expression patterns (Swanson-Wagner et al., 2012). Included among the 600 were a mere 46 that had been previously identified as targets of selection and a similar number presumed to result as an effect of inbreeding.

Similarly to the above-described maize study, research in tomato used RNA-seq to define both gene sequence and expression divergence between cultivated tomato and five related wild species (Koenig et al., 2013). Based on sequence differences, Koenig et al. (2013) detected footprints of positive selection in over 50 genes and documented thousands of shifts at the gene-expression level. These rapidly evolving genes are commonly associated with environmental responses and stress tolerance. Similarly, decreased nucleotide and expression diversity and modified co-expression patterns characterized the domestication of common bean (Bellucci et al., 2014). Moreover, RNA sequencing of 240 accessions of lettuce revealed a list of regions as putative selective sweeps that occurred during domestication and divergence, respectively (Zhang et al., 2017). Genome-wide association studies (GWASs) further identified 5,311 expression quantitative trait loci (eQTL) regulating the expression of 4,105 genes, including nine eQTLs regulating genes associated with flavonoid biosynthesis and thereby underlying color and nutritional content of the crop. Indeed, the GWAS approach is highly useful alongside the selective sweep approach in characterizing changes on domestication. While there are variants as to the mathematical model underlying such analyses, they are all essentially highly similar in that they associate genotypic and phenotypic variance (lists of GWAS databases and commonly used approaches are provided in Tables 1, 2).

**Table 1:** List of database and tools commonly used for GWAS and genomic studies

Name	Link	Refernce
easyGWAS: a cloud-based platform for comparing the results of genome-wide association studies	https://easygwas.ethz.ch/	Grimm et al. (2017)
Matapax: an online high-throughput genome-wide association study pipeline	https://matapax.mpimp-golm.mpg.de/	Childs et al. (2012)
GWAPP: a web application for genome-wide association mapping in Arabidopsis	https://gwas.gmi.oeaw.ac.at/	Seren et al. (2012)
GWAS Atlas: a curated resource of genome-wide variant–trait associations in plants and animals	https://bigd.big.ac.cn/gwas/	Ye et al. (2020)
PGSB PlantsDB: updates to the database framework for comparative plant genome research	https://pgsb.helmholtz-muenchen.de/plant/index.jsp	Spannagl et al. (2016)
Phenotypic and genome-wide association with the local environment of Arabidopsis	http://www.personal.psu.edu/sma3/CLIMtools.html	Ferrero-Serrano and Assmann (2019)
RiceVarMap: a comprehensive database of rice genomic variations	http://ricevarmap.ncpgr.cn/v2/	Zhao et al. (2014)
GWASpro: a high-performance genome-wide association analysis server	https://bioinfo.noble.org/GWASPRO/	Kim et al. (2018)
MaizeGDB 2018: the maize multi-genome genetics and genomics database	https://www.maizegdb.org/	Kim et al. (2018)
TASUKE+: a web-based platform for exploring GWAS results and large-scale resequencing data	https://tasuke.dna.affrc.go.jp/	Kumagai et al. (2019)
ZEAMAP, a comprehensive database adapted to the maize multi-omics era	http://www.zeamap.com	Gui et al. (2020)
MaizeCUBIC: a comprehensive variation database for a maize synthetic population	http://modem.hzau.edu.cn/	Luo et al. (2020)
CARMO: a comprehensive annotation platform for functional exploration of rice multi-omics data	http://bioinfo.sibs.ac.cn/carmo	Wang et al. (2015)
AraQTL—workbench and archive for systems genetics in *Arabidopsis thaliana*	http://www.bioinformatics.nl/AraQTL/	Nijveen et al. (2017)
WheatExp: an RNA-seq expression database for polyploid wheat	http://wheat.pw.usda.gov/WheatExp/	Pearce et al. (2015)
CerealsDB 2.0: an integrated resource for plant breeders and scientists	https://www.cerealsdb.uk.net/cerealgenomics/CerealsDB/indexNEW.php	Wilkinson et al. (2012)
The Triticeae Toolbox: combining phenotype and genotype data to advance small-grains breeding	http://triticeaetoolbox.org	Blake et al. (2016)
The AraGWAS Catalog: a curated and standardized *Arabidopsis thaliana* GWAS catalog	https://aragwas.1001genomes.org	TogninalLi et al. (2018)
Gramene: a resource for comparative analysis of plants genomes and pathways	http://www.gramene.org	Tello-Ruiz et al. (2018)
Ensembl Genomes 2020—enabling non-vertebrate genomic research	http://www.ensemblgenomes.org	Howe et al. (2020)
SnpHub: an easy-to-set-up web server framework for exploring large-scale genomic variation data in the post-genomic era with applications in wheat	http://wheat.cau.edu.cn/Wheat_SnpHub_Portal/	Guo et al. (2020)

**Table 2:** Commonly used packages for conducting GWAS^a^

Package	Description	Web page
TASSEL	Variety of algorithms MLM, GLM, weighted MLM, genomic selection, fast association; supports P3D compression; can process GBS data; designed to determine dominance/additivity of effects; user‐friendly GUI	http://www.maizegenetics.net/tassel
GAPIT	Package that can perform MLM and EMMA; supports P3D and EMMAx; works via R language	http://www.maizegenetics.net/gapit
A-DTest	R package is ADGWAS: for GWAS	https://github.com/maizego/A-D-test
EMMAX	Efficient mixed‐model method for large genomic datasets; command‐line interface only	http://genetics.cs.ucla.edu/emmax/index.html
GEMMA	Standard/multivariate/Bayesian linear mixed‐model framework; estimates quantitative genetic traits and proportioning of variance; command‐line interface only	http://www.xzlab.org/software.html
ANGSD	Useful when genotypic states are not known with certainty; measures population genetic parameters; command‐line interface only	http://www.popgen.dk/angsd/index.php/ANGSD
Plink	Wide‐ranging toolset for conducting GWAS; originally designed for human genome data; command‐line interface only	http://pngu.mgh.harvard.edu/˜purcell/plink/
Lrgpr	Allows for testing of G × G and G × E; works via R language	http://lrgpr.r‐forge.r‐project.org/

a Modified from Burghardt et al. (2017).

A wide range of bitter-tasting compounds were selected against during the processes of domestication and improvement including the steroidal glycoalkaloids of tomato and potato (*Solanum tuberosum*; Itkin et al., 2013; Schwahn et al., 2014), β-L-oxayl-2,3-diaminopropionic acid (β-L-ODAP) in grass pea (Emmrich et al., 2019), curcurbitadienol in cucumber (*Cucumis sativus*; Shang et al., 2014; Zhou et al., 2016), glucosinolates in broccoli (*Brassica oleracea*; Drewnowski and Gomez-Carneros, 2000), and the flavone-7-*O*-neohesperidoside in citrus (Frydman et al., 2013). While the decrease in the levels of these metabolites is rather predictable and moreover easy to rationalize metabolome wide studies in wheat (Beleggia et al., 2016), maize (Xu et al., 2019), rice (Deng et al., 2020), and tomato (Zhu et al., 2018) and latterly in lettuce (Zhang et al., 2020) and tea (Zhang et al., 2020) revealed far more complex changes.

In the first of these studies, Beleggia et al. (2016) showed that the primary domestication of wheat was characterized by a reduction in unsaturated fatty acids on the primary domestication with altered amino acid content characterizing the secondary domestication. Maize, by contrast was characterized by alkaloid, terpenoid, and lipid changes at the divergence between teosinte and tropical maize, whereas benzoxazinoid levels changed at the divergence between tropical and temperate maize (Xu et al., 2019) and rice displayed different changes again (Deng et al., 2020). Tomato domestication had highly diverse effects on the metabolome, with many metabolic changes being associated with the increase in size, others with breeding for color preferences and yet further by the introgression of disease resistance from wild relatives (Zhu et al., 2018). Likewise in lettuce, quinate and chlorogenic acid levels were strongly reduced on domestication of lettuce, probably as a consequence of the desire to reduce bitterness (Zhang et al., 2020). By contrast tea, probably as a result of its complex domestication, did not display clear changes in its metabolite content across the domestication and improvement processes (Zhang et al., 2020).

While transcriptomics and metabolomics and corresponding GWAS analysis can clearly allow the computational discrimination of domestication genes, the number and scale of changes at the transcript and metabolite level suggest that transcriptomics and metabolomics will be necessary for the regulatory control of any future de novo domesticated product. Such control has been debated for CRISPR/Cas lines (Fedorova and Herman, 2020; Fraser et al., 2020) in general but will be particularly important for de novo domestication events (either using CRISPR/Cas or conventional breeding) using species that are currently not eaten in great amounts in order to ensure their safety. For this purpose, a comparison of the metabolomes of de novo domestication events against conventional crops will prove highly worthwhile, although it will be important to improve the curation of such a comparison on our current knowledge concerning the (anti)nutritional values of the individual metabolites in our major crops such that this is as good as the FAO yield statistics (http://www.fao.org).

## The use of precision phenotyping to demonstrate the phenotypic scope of de novo domestication

As demonstrated above, a pipeline for identifying target genes for de novo domestication is relatively easy following analysis of our current crops and their wild relatives. Indeed, a number of key genes for improvement in orphan crops have previously been discussed, including genes involved in plant architecture (barley *SEMI-DWARF1*, *TEOSINTE BRANCHED1*, *DEEPER ROOTING1*, *PHOSPHORUS-STARVATION TOLERANCE1*, *PROG1*; Jia et al., 2009; Uga et al., 2013; Mai et al., 2014; Shang et al., 2014; Studer et al., 2017), seasonal flowering time (*PHOTOPERIOD-H1*, *CENTRORADIALIS*; Turner et al., 2005; Comadran et al., 2012), light competition (*PHYB1* and *PHYB2*; Sheehan et al., 2007), seed or fruit retention (*SHATTERING1* and others; Lin et al., 2012; Meyer and Purugganan, 2013), fruit size (*FRUIT WEIGHT2.2*; Frary et al., 2000), and length of the juvenile stage (*TERMINAL FLOWER 1*, *FLOWERING LOCUS T*; Bergonzi and Albani, 2011; Yamagishi et al., 2014). Similarly, as the proof-of-concept studies described above demonstrate, targeted manipulation of multiple genes can be readily carried out. That said, selecting which relative is the best choice to target for the process is considerably more difficult.

**Figure 2:**
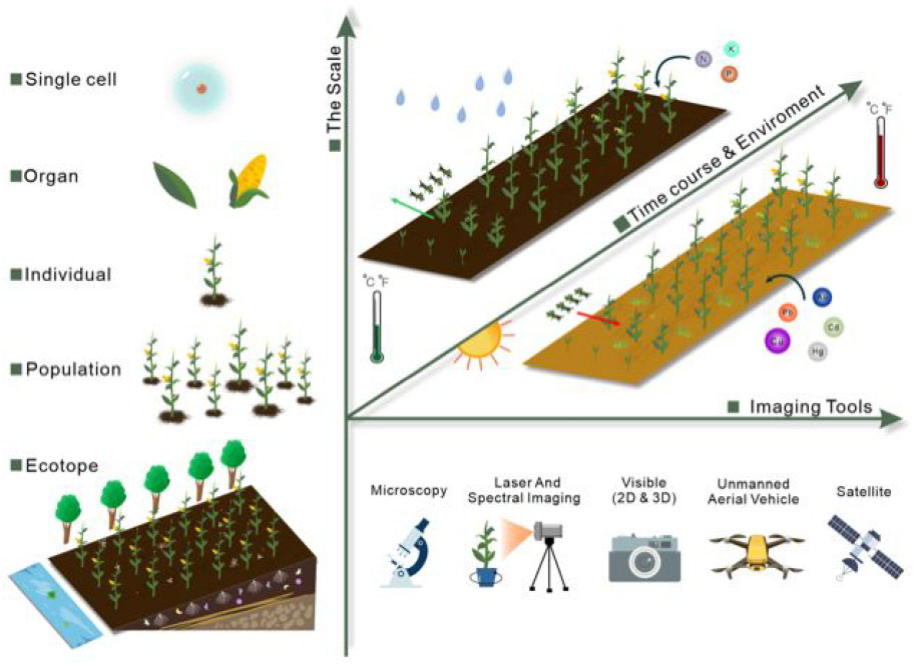
Utilizing multiple crop phenotyping tools for accurate high-throughput acquisition and analysis of multidimensional phenotypes on organism-wide scale (from single cell to ecotype). Examples range from micro to macro scale, through different environments (abiotic stress, biotic stress, etc.) and across the entire crop developmental process.

**Figure 3:**
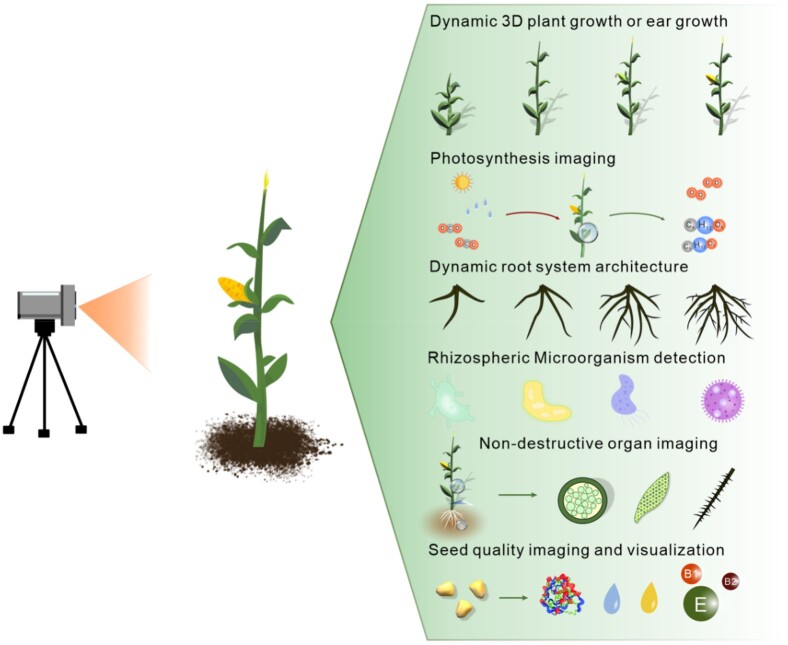
The utilization of crop phenotyping techniques. They can be applied widely for example in dynamic measurements of 3D plant growth, ear growth, photosynthesis, root system architecture, rhizospheric microorganism detection, non-destructive organ imaging, and the visualization of seed quality distribution.

We believe that this question is best resolved by comparative phenotyping of a range of species of local wild species against the major cultivated crop as well as select genotyping of the level of allelic variance in the domestication gene in question. To the best of our knowledge, such experiments, while perhaps underway, have not yet been published. We did, in a previous article, suggest a range of non-cultivated and semi-cultivated relatives (Fernie and Yan, 2019) and this list still stands. However, it is important to note that not only are these species under-utilized but they are also understudied. Indeed, the same could be held true until recently even for major crops such as cassava, sweet potato, and yam as well as nutritionally important crops such as quinoa. The recent publication of the genomes of these species (Wang et al., 2014; Jarvis et al., 2017; Yang et al., 2017; Scarcelli et al., 2019) alongside considerably better characterization of them at the physiological and metabolic levels (Obata et al., 2020; Price et al., 2020; Sonnewald et al., 2020) provides an effective blueprint as to how candidates for de novo domestication should be assessed. We summarize the set of tools that we believe should be brought to bear in field trials comparing the effects of established crops and candidates for de novo domestication in Figure 2. Here, we suggest that it will be important to use contemporary technology to consider all levels of the spatial hierarchy from single cells (Luo et al., 2020) to ecosystem models (Tian et al., 2020), using a broad range of imaging tools to record and also infer trait variances (Yang et al., 2020) In this vein, the power of inferential data has been provided by a recent proof of concept study comparing hyperspectral imaging of metabolite content to measurements made by mass-spectrometry (Vergara-Diaz et al., 2020) implying that this may shortly be as reliable as spectral measurements of photosynthesis from unmanned aerial vehicles have proven to be (Gago et al., 2020; Yang et al., 2020). Indeed, the data emanating from such imaging platforms (and those presented in Figure 3) will certainly form an essential component of deciding which species would be ideal for any given environment for the purposes of de novo domestication. As an extension to this strategy, the use of the reciprocal transplant strategy (Hereford, 2009; Ågren and Schemske, 2012; Sork, 2018), whereby species are planted and monitored in the environment of one another in addition to their own, would allow the adaptation of exotic species to be quantified in a human-controlled environment as a first indicator that aids in the selection of the best candidates for de novo domestication.

As we state above, to our knowledge, public data are not currently available for comparison of domesticated and non-domesticated species. However, all of these phenotyping methods have been demonstrated to provide reliable information (Watt et al., 2020; Yang et al., 2020). As such we would envisage the use of such precision phenotyping approaches, in comparative analyses, to be instrumental in narrowing down the lists mentioned above and beginning to define ideal targets for de novo domestication. This is very easy to state but how do we propose that this will work in practice. Well for starters examination of certain key parameters could be used for example the rate of photosynthesis, ease of harvest, productivity, abiotic resilience, biotic resistance, ability to prosper in a dense stand, etc. Such traits could be computationally afforded different weighting with regard to the most challenging aspects of the environment the novel crop was planned for thereby providing a ranking of candidate species for a given environment. At a practical level, the complexity of obtaining the genome sequence and/or of generating allelic variants in the gene(s) of interest (via either gene editing or introgression) would likely considerably narrow these lists. However, despite current limitations, genome sequencing solutions for complex genomes are becoming available and as we detail below considerable advances are also being made in transformation of species that were previously regarded as recalcitrant.

## The power of CRISPR/Cas libraries

Following the selection of the candidate species for de novo domestication, two further hurdles remain. First, it would be prudent to have the genome of the species to be domesticated in hand such that one could be sure that the genetic interventions planned were sensible. Next-generation sequencing technologies have rendered this relatively facile and even large polyploidy genomes can be tackled without trepidation. Second, on having identified the genetic targets to modify a route for genetic manipulation is needed. For this purpose, two clear possibilities exist. Using classical breeding, genes of interest can be introgressed into the candidate for de novo domestication providing that the source of these genes is closely genetically related to the recipient. Alternatively, chemical mutagenesis or the genome editing approach can be used. The use of the latter approach is complicated by the fact that regulatory concerns will render agricultural production of plants created by such technologies difficult in certain countries. However, given that it is more rapid, and more precise we will concentrate on this approach here. While Zinc Finger Nucleases and Transcription Activator like effector nucleases are also utilized, Clustered Regularly Interspaced Short Palindromic Repeats/CRISPR-associated protein (CRISPR-Cas) has moved to the fore primarily due to its simplicity and efficiency. A great advantage of the CRISPR/Cas system is that it can be used to edit multiple genes simultaneously (Xie and Yang, 2013; Zhang et al., 2014; Ma et al., 2015; Qi et al., 2016), and can generate large mutant libraries in a scalable high-throughput manner (Lu et al., 2017; Meng et al., 2017; Liu et al., 2020).

CRISPR-Cas systems have already shown their superiority in precision breeding through editing coding regions and knocking out large numbers of genes of interest (reviewed in Chen et al., 2019; Liu et al., 2021). However, fine-tuning of the expression of target genes via editing cis-regulatory sequences or changing the status of epigenetic marks is also very promising for future breeding designs (Liu et al., 2021). It is particularly worth mentioning that the further application of big data analysis methods utilizing machine-learning approaches may enable us to accurately understand and predict the function of each gene, segment of sequence, or even each base. Combined with CRISPR technology, not only we can accurately modify target sequences, but also can create new sequences, thereby altering their function(s). This will render future de novo domestication both more efficient and more accurate (Liu et al., 2020). In this vein, Rodríguez-Leal et al. (2017) provided a seminal example of obtaining quantitative variation in the important agronomic traits fruit size, inflorescence branching, and plant architecture via gene editing. While this was carried out in a domesticated species as a proof-of-concept, it is clearly conceivable that introducing such common domestication traits to underutilized species may well improve their prospects as agricultural commodities.

As mentioned above, a major constraint in our ability to harness the potential of other species is the difficulty in producing the allelic variance. While in many cases this goal could be achieved by mutagenesis or gene introgression via classical breeding, such approaches are relatively slow. Therefore, expanding the number of species that can be transformed is of vast importance. Recent studies have reported considerable improvements in the efficiency of plant regeneration from tissue culture are achievable by overexpression of plant developmental regulators including *LEAFY COTYLEDON1* or *LEAFY COTYLEDON2* (Lotan et al., 1998; Stone et al., 2001), *WUSCHEL* (Zuo et al., 2002), or *BABY BOOM* (Boutilier et al., 2002)*.* Moreover, a recent study has shown that a fusion protein combining wheat *GROWTH-REGULATING FACTOR4* and its cofactor *GRF-INTERACTING FACTOR1* substantially increases the efficiency and speed of regeneration in wheat, triticale, and rice and increases the number of transformable wheat genotypes (Debernardi et al., 2020). These examples, alongside that of the transformation of the wild rice *O. alta* would appear to offer great promise for this approach. However, the ability to introduce the planned modification into the genome of the potential novel crop—be it by classical, mutagenesis, or gene editing approaches—should not be underestimated as it is currently the major challenge facing the de novo domestication approach. A couple of recent developments, in addition to those mentioned above offer hope here though. The first is the development of efficient grafting methods that would allow wild species to act as root-stocks as a route to novel agricultural products. This approach has often been discarded due to graft incompatibilities, but this challenge has been overcome in many crops (Notaguchi, 2020). The second is the recent finding by the group of Dan Voytas that gene-edited dicots can be generated via de novo meristem induction (Maher et al., 2020). In this method, developmental regulators and gene editing reagents can be delivered to somatic cells in order to generate inheritable changes in sequence via a route that bypasses tissue culture. The development of such approaches thus provides optimism that in the future the challenge of modifying lesser grown plants could prove less daunting.

Despite the immense interest in these approaches, there are some concerns about the application of the CRISPR-Cas system in crop breeding, the biggest of these being the possibility of deleterious effects caused by the integration of transgenic constructs or off-target mutations. Several studies have documented the off-target effects of the CRISPR-Cas system in plants (Xie and Yang, 2013; Zhang et al., 2014; Endo et al., 2015; Jacobs et al., 2015; Jin et al., 2019). Computational analysis of the likelihood of off-target effects is a pre-requisite for precise de novo domestication approaches. In parallel, other methods of assessing genome edited crops such as at the level of the metabolome have been proposed as methods of ensuring that unintended effects of the editing can be monitored and, if these are negligible, that such crops can be regarded as safe (Fraser et al., 2020). Such analyses are important for all new crops; however, they will be far more important for true do novo domesticates as opposed to minor crops that are already consumed such as members of the amaranth genus (Stetter et al., 2020), or fonio millet (*Digitaria exilis*; Abrouk et al., 2020). It can only be hoped that a combination of such approaches, alongside the publicity generated by the 2020 Nobel Prize for Chemistry will allay public skepticism of gene editing.

One aspect that is difficult to envisage being tractable by gene editing is the harnessing of trait variation resulting from natural structural genome variants (Alonge et al., 2020; Alseekh et al., 2020; Domínguez et al., 2020; Fraser et al., 2020). It is becoming apparent from resequencing and the assembly of pan-genomes that structural variants beyond the mere addition or deletion of genes play important roles in shaping crop phenotypes. The results of two recent studies in tomato are particularly pertinent here that of Domínguez et al. (2020) revealed the importance of transpostional insertion on transcription with potential consequences on virus and Phytophthora resistance as well as shelf life while GWASs revealed structural variants associated to a number of these (Domínguez et al., 2020). Similarly, the study of Alonge et al. (2020) identified a P450 gene duplication underlying a fruit weight quantitative trait loci (which was previously thought to be associated with a single nucleotide polymorphism (SNP)) and other structural variants, which were required for breeding of the jointless trait (Alonge et al., 2020). Adoption of introgression-based strategies would be one current approach to harness such variation. However, whether such approaches will be necessary awaits further, more detailed analysis of structural genome variants in the majority of our crop species. That said, it is important to note here that the CRISPR/Cas9 has already been demonstrated to display the diversity required to address these questions in plants (Li and Xia, 2020). In particular, it has been shown to induce chromosomal translocations (Beying et al., 2020), large inversions (Schwartz et al., 2020), and to change recombination patterns (Schmidt et al., 2020).

## The prospects of integrating molecular markers and metabolites into models to improve phenotype prediction

Several other computational tools warrant discussion within the context of de novo domestication, including prediction of changes in the levels of molecular and morphological aspects of phenotype. A relatively simple example that is pertinent in this context is the recent attempt to model gene expression of the phenylpropanoid pathway a wide range of wild species tomato on the basis of metabolome data from the same samples (Tohge et al., 2020) with this being the culmination of several years of experience in integrative analyses reviewed in a previous Update in Plant Physiology (Tohge et al., 2015). Something that has proven considerably more complex is the prediction of yield from metabolomics data. This has nevertheless been attempted (Meyer et al., 2012; Riedelsheimer et al., 2012; Rosado-Souza et al., 2015). While generally speaking it is difficult to find a single metabolite whose level is predictive of yield, a wide number of studies have revealed metabolic signatures for this in a range of species including Arabidopsis, maize, wheat, and tomato (Schauer et al., 2006; Meyer et al., 2012; Riedelsheimer et al., 2012; Obata et al., 2015; Vergara-Diaz et al., 2020). Application of such tools to de novo domesticated crops will be a highly useful strategy to search for additional manipulations that will allow further improvement of these novel crops.

In addition, genomic selection approaches that utilize molecular markers and machine learning to identify superior genotypes with improved traits such as growth have started to incorporate –omics level data. Given that this topic has recently been comprehensively reviewed, we do not discuss it in detail here (Tong and Nikoloski, 2020). Suffice to say that results from a recent study suggest that integrating molecular markers into metabolic models can dramatically improve the prediction accuracy of genomic selection strategies (Tong et al., 2020). We thus feel it likely that the integration of such extended models, as well as the integration of other machine learning strategies such as those described in Liu et al. (2020) will most likely prove highly informative in the development of second generation de novo domesticates.

## Conclusion and future perspectives

As we have described above, a growing number of examples have demonstrated that it has become relatively facile to de novo domesticate plants. Indeed, technological advances in nucleotide sequencing and gene editing have greatly expanded our capability to tackle such ambitious projects. We argue here that given these stunning advances, one of the most difficult aspects of this procedure will be the choice of a suitable starting species presenting a potential route by which to address this question. Multiple roles for computation can be envisaged in the process of de novo domestication from comparative phenotypic evaluation of potential starting species, through genome analysis and population genomics to the comparative evaluation of de novo domesticates and their progenitors. The need for computation in the design of CRISPR/Cas strategies, the evaluation of GWASs, multi-omics integration, genomic prediction, and deep learning is equally pervasive. Indeed, with the increasing sophistication of available genotyping and phenotyping data, it is becoming harder and harder to imagine any large-scale de novo domestication program not employing the tools of precision phenotyping. Despite its great promise, a number of important and even fundamental questions remain as yet unanswered (see Outstanding Questions Box). Moreover, considerable challenges that limited and constrained crop domestication over millennium (Stetter, 2020) will ultimately also need to be considered when approaching de novo domestication. Among these the genetic architecture underlying traits, the level of standing genetic variation for domestication traits, and the accumulation of genetic load (i.e. deleterious genetic variants) have been noted as important factors determining the extent of domestication of various species. Less domesticated crops are often well adapted to diverse environments and of high nutritional value but need improvement in key domestication traits to render them serious alternatives to our existing crops. We believe that the key steps in de novo domestication are three-fold: (i) the comprehensive evaluation of a wide range of lesser grown crops alongside the acquisition of (ii) high-quality genome sequences, (iii) knowledge concerning functional genes, and (iv) the competence to generate variance in key genes of interest. While the examples to date suggest that such approaches are possible, a lot of research and development will be required before they become routine.

**Outstanding questions box**

- Where is our greatest need for de novo domestication?
- What are the most suitable species to act as progenitors to the de novo domesticates?
- Can we improve minor crops with the tools of de novo domestication?
- Can we tailor the generation of de novo domesticates to perform better in projected future climates?
- Will it be possible to utilize deep-learning methods to improve future strategies?

## Notes

A.R.F. and J.Y. designed the review concept. All authors wrote the manuscript and finally approved the final version of the manuscript.

The author responsible for distribution of materials integral to the findings presented in this article in accordance with the policy described in the Instructions for Authors (https://academic.oup.com/plphys/pages/general-instructions) are: Alisdair R. Fernie (fernie@mpimp-golm.mpg.de) and Jianbing Yan (yjianbing@mail.hzau.edu.cn).
